# Effect of vitamin D supplementation on 24-hour urine calcium in patients with calcium Urolithiasis and vitamin D deficiency

**DOI:** 10.1590/S1677-5538.IBJU.2018.0522

**Published:** 2019-04-01

**Authors:** Maryam Taheri, Sanaz Tavasoli, Fatemeh Shokrzadeh, Fahimeh Bagheri Amiri, Abbas Basiri

**Affiliations:** 1 Urology and Nephrology Research Center, Shahid Beheshti University of Medical Sciences, Tehran, Iran

**Keywords:** Parathyroid Hormone, Urolithiasis, Vitamin D

## Abstract

**Purpose:**

Hypercalciuria is one of the risk factors for calcium kidney stone formation (the most common type of urinary stones). Although vitamin D deficiency is prevalent among urolithiasis patients, the effect of vitamin D supplementation on urine calcium in these patients is still unclear.

**Materials and Methods:**

In this retrospective study, medical and laboratory tests records of 26 patients with recurrent calcium kidney stones and vitamin D deficiency treated with 50000IU vitamin D per week for 8-12 weeks were analyzed. The changes in 24-hour urine calcium (24-h Ca), serum 25-hydroxyvitamin D (25 (OH) D), serum parathormone (PTH), other 24-hour urine metabolites and calculated relative supersaturations of calcium oxalate (CaOxSS), calcium phosphate (CaPSS) and uric acid (UASS) were assessed. Moreover, correlations between changes in 24-h Ca and other aforementioned variables were assessed.

**Results:**

Serum 25 (OH) D and 24-h Ca increased after vitamin D supplementation, while serum PTH decreased (p < 0.001, for all analyses). The levels of 24-hour urine sodium and urea increased significantly (p = 0.005 and p = 0.031, respectively). The levels of CaOxSS and CaPSS increased, but the changes were not significant (p = 0.177, and p = 0.218, respectively). There were no correlations between the changes in 24-h Ca and serum 25 (OH) D or PTH.

**Conclusions:**

The result of current study suggests that although urine Ca increased in vitamin D supplemented patients, this increase was not associated with the increase in serum vitamin D and may be due to other factors such as dietary factors. Further randomized clinical trials considering other factors associated with urine Ca are warranted.

## INTRODUCTION

The urinary stone disease is a common disease with a high risk of recurrence and increasing prevalence around the World (1). Calcium (Ca) stones are the most common type of urinary stones in many countries, including Iran (2). Hypercalciuria is the most common risk factor for Ca kidney stone in many countries (3). Many factors including non-dietary and dietary factors are shown to cause hypercalciuria; however, the exact impact of some of these factors on urinary Ca excretion is still unclear (4).

Vitamin D deficiency is one of the most common health problems all over the World. Studies have shown a high prevalence of moderate to severe vitamin D deficiency in various cities of Iran (5). Because of the essential role of vitamin D in Ca homeostasis and bone health, as well as its role in different chronic diseases (6, 7), many guidelines emphasize the importance of treating vitamin D deficiency (8, 9).

According to current studies, vitamin D deficiency is prevalent among patients with kidney stones (10–12). However, the treatment of vitamin D deficiency in kidney stone patients is a matter of debate (13), according to the high prevalence of osteoporosis and low bone density (14), and the limited and conflicting results regarding the effect of vitamin D supplementation on the risk of developing hypercalciuria in patients with kidney lithiasis.

The current study aims to evaluate the effect of vitamin D supplementation on 24-hour urine Ca in a group of patients with recurrent Ca kidney stones and vitamin D deficiency.

## MATERIALS AND METHODS

### Study Design and patients

In this retrospective study, the medical records of patients referred to the Stone Prevention Clinic of Labbafinejad Hospital, Tehran, Iran, in recent two years (December 2015 to December 2017) were reviewed. Patients with history of recurrent kidney stones (with a history of at least two radiopaque stone episodes) (15) and vitamin D deficiency [serum 25 hydroxyvitamin D (25 (OH) D) below 30ng / mL] who were treated with 50000IU vitamin D per week for 8-12 weeks, according to patient's demographic and anthropometric data (8), were enrolled. Other inclusion criteria were baseline 24-hour urine Ca below 300mg / 24hrs; age over 18 years and existence of valid data of serum 25 (OH) D and 24-hour urinary analysis before and after vitamin D supplementation. Participants were excluded if they meet at least one of the following criteria: known history of diabetes mellitus; primary hyperparathyroidism; malignancy or malabsorption; any disease that affects serum 25 (OH) D and Ca or 24-hour urine Ca (such as sarcoidosis and some other chronic granulomatous disorders); using other forms of vitamin D supplement (lower doses or intramuscular) during the treatment period; changes in dosage or new addition of thiazide diuretics, or any drug which may affect serum or urine Ca during the treatment period (such as lithium); taking Ca supplements; history of vitamin D supplementation (oral or intramuscular) three months prior to study; 24-hour urine under-collection (24-hour urine creatinine (Cr) > 800mg for men and > 600mg for women (16)); and pregnancy or lactation. All patients received nutritional advice for the prevention of kidney stone according to European Association of Urology (EAU) guideline (17).

### Study outcomes and laboratory tests

Patient information such as demographic and anthropometric data, past medical and drug history had been collected by physicians of Stone Prevention Clinic of Labbafinejad Hospital, as previously described (18).

Fasting blood samples were taken from patients and blood serums were separated for the analyses. The 24-hour urine samples were collected with hydrochloric acid 6N as the preservative. Serum 25 (OH) D and parathormone (PTH) were measured by the electrochemiluminescent method (Elecsys 2010 automatic analyzer, Roche Hitachi). Serum or urine urea, Cr, Ca, phosphorous, sodium (Na), potassium, uric acid, magnesium (Mg), citrate (Cit) and oxalate (Ox) concentrations were analyzed as reported previously (19). Relative Supersaturations of CaOx (CaOxSS), Ca phosphate (CaPSS) and uric acid (UASS) were calculated using LithoRisk software (Biohealth, Italy) (20), using measured 24-hour urine metabolites.

### Statistical analyses

All data analyses were performed using SPSS version 23. Normality of data was checked by Shapiro Wilk test and Q-Q plot. Within-group differences were assessed by a Paired-Samples T test (for normally distributed continuous data) and Wilcoxon signed-rank test (for Skewed continuous data). Correlations between changes in 24-h Ca (24-h Ca diff) and changes in serum 25 (OH) D (25 (OH) D diff), changes in serum PTH (PTH diff) and changes in other 24-hour urine metabolites were assessed. Pearson correlation coefficient was used for normally distributed continuous data and Spearman correlation coefficient was used for skewed continuous data. The level of significance was set at p-value < 0.05.

## RESULTS

From 334 kidney stone patients who were treated for vitamin D deficiency at the time of the study, 26 met the inclusion and exclusion criteria and enrolled in the analyses. The most common reason for exclusion was taking a thiazide diuretic. Baseline characteristics of patients are shown in Table-1. The majority of participants had serum 25 (OH) D in the range of 10-19ng / mL. Seven patients (26.9%) had baseline hyperparathyroidism (PTH > 65pg / mL).

**Table 1:** Baseline characteristics of recurrent calcium stone patients with vitamin D deficiency. All values are mean (SD) unless otherwise mentioned.

		Value
Gender (male%)		20 (76.9%)
Age (years)		47.5 (12.31)
BMI (a) (kg / m^2^)		28.00 (5.13)
Follow-up period (month) [median (IQR)]		4.12 (2.20)
Serum BUN (b) (mg / dL)		14.58 (3.42)
Serum Creatinine (mg / dL)		1.15 (0.14)
Serum calcium (mg / dL)		9.65 (0.45)
Serum phosphorus (mg / dL)		3.26 (0.58)
**25-hydroxyvitamin D (ng / mL) n (%)**		
	0-9	6 (23.1%)
	10-19	15 (57.7%)
	20-25	5 (19.2%)
**PTH** (c) **(pg / mL) n (%)**		
	< 65	19 (73.1)
	≥ 65	7 (26.9)

(a) = Body Mass Index;

(b) = Blood Urea Nitrogen;

(c) = Parathormone

Urine and serum parameters before and after vitamin D supplementation (median follow-up period of 4.12 months) are shown in Table-2. The mean level of serum 25 (OH) D increased significantly after vitamin D supplementation (p < 0.001). A detailed overview showed that the level of 25 (OH) D did not increase in 2 patients (7.7%) and did not reach the normal levels (> 30) in 9 patients (34.6%). The level of 24-h Ca increased and the level of serum PTH decreased significantly after vitamin D supplementation (p < 0.001, for both analyses). Considering other 24-hour urine metabolites, the levels of 24-hour urine Na (24-h Na) and Urea (24-h Urea) increased significantly (p = 0.005 and p = 0.031, respectively). The levels of CaOxSS and CaPSS also increased, but the changes were not significant (p = 0.177, and p = 0.218, respectively) (Table-2).

**Table 2:** Urine and serum parameters before and after vitamin D supplement therapy. All values are mean (SD) and p-value stands for the paired t-test unless otherwise mentioned.

	Before	After	P value
25 (OH)D (ng / mL) (n = 26)	14.08 (5.49)	33.64 (13.89)	< 0.001***
Serum PTH (pg / mL) (n = 19)	55.89 (21.93)	38.42 (15.39)	< 0.001***
Serum Ca (mg / dL) (n = 18)	9.66 (0.44)	9.74 (0.35)	0.537
24-hour urine Ca (mg / day) (n = 26)	149.92 (78.61)	229.92 (104.83)	< 0.001***
24-hour urine Na (mEq / day) (n = 26)	133.89 (51.37)	171.08 (54.65)	0.005**
24-hour urine Urea (gr / day) (n = 19)	22.97 (8.03)	28.74 (12.19)	0.031*
24-hour urine UA (mg / day) (n = 26)	413.67 (135.22)	455.35 (194.15)	0.357
24-hour urine P (gr / day) † (n = 26)	0.71 (0.30)	0.78 (0.36)	0.08
24-hour urine K (mEq / day) † (n = 26)	50.58 (20.80)	60.66 (29.27)	0.027*
24-hour urine OX (mg / day) (n = 26)	34.74 (17.68)	36.63 (17.54)	0.705
24-hour urine Cit (mg / day) (n = 26)	534.5 (252.2)	517.2 (281.4)	0.715
24-hour urine Mg (mg / day) † (n = 26)	78.08 (37.72)	113.08 (50.52)	0.003**
24-hour urine Cr (mg / day) (n = 26)	1.23 (0.46)	1.28 (0.44)	0.156
24-hour volume (mL) † (n = 26)	2000.6 (883.7)	2190.4 (791.6)	0.306
CaOX supersaturation (n = 26)	4.675 (2.652)	5.394 (3.202)	0.177
CaP supersaturation † (n = 26)	0.332 (0.297)	0.568 (0.722)	0.218
UA supersaturation (n = 26)	1.213 (0.922)	1.155 (0.860)	0.765

25 (OH) D = 25-hydroxyvitamin D; PTH = parathormone; Ca = calcium; Na = sodium; UA = uric acid; P = phosphorus; K = potassium; Ox = oxalate; Cit = citrate; Mg = magnesium; Cr = creatinine; CaOx = calcium oxalate; CaP = calcium phosphate

† P value stands for Wilcoxon signed-rank test

* p<0.05;

** p<0.01;

*** p<0.001

**Figure 1:**
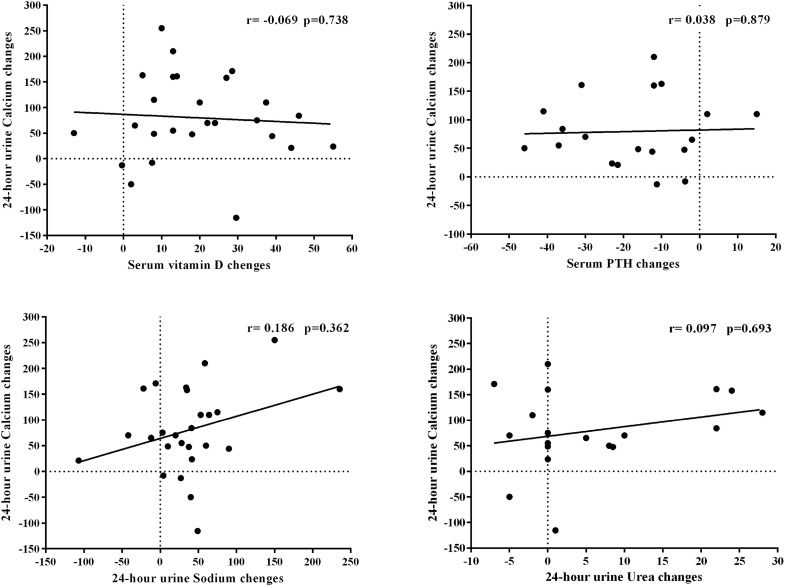
The correlation between the changes in 24-hour urine calcium (24-h Ca) and the changes in serum 25 (OH) D, serum PTH, 24-hour urine sodium (24-h Na) and urea (24-h Urea).

The correlation between 24-h Ca diff and changes in other variables were assessed. There was no correlation between 24-h Ca diff and baseline serum 25 (OH) D level (r = 0.175, p = 0.392), 25 (OH) D diff (r = −0.069, p = 0.738), or PTH diff (r = 0.038, p = 0.879) (Figure-1). Moreover, there was no correlation between 24-h Ca diff and the changes in 24-h Na (24-h Na diff) (r = 0.186, p = 0.362), changes in 24-h Urea (24-h Urea diff) (r = 0.097, p = 0.693) or changes in other variables.

## DISCUSSION

The results of the current study indicate that, although urine Ca increased in vitamin D supplemented patients, this increase was not associated with the increase in serum vitamin D. There are limited trials, which assessed the effect of vitamin D supplementation on urine Ca in patients with kidney stones. To the best of our knowledge, only three studies had evaluated this effect (21–23). In a study by Leaf et al. (21), 29 patients with renal stones received 50.000IU vitamin D per week for eight consecutive weeks. In a study by Ferroni et al. (22), patients received either 1.000IU vitamin D daily (n = 8) or 50.000IU vitamin D per week (n = 13) for 6 weeks. Finally, Hesswani et al. (23) retrospectively evaluated 34 patients with renal stones, which were treated with a median intake of 1.000IU vitamin D, and 945mg Ca supplements simultaneously. The median time of follow-up was 39 months. The only study that reported an increase in urinary Ca was the study by Hesswani et al., in which patient received vitamin D and Ca co-supplements. However, in line with our results, there was no correlation between the changes in urinary Ca and changes in serum vitamin D in the study by Hesswani et al. The authors conclude that simultaneous consumption of Ca supplement may be a cause of urinary Ca increase. A meta-analysis by Malihi et al. (24) studied the effect of vitamin D and Ca co-supplementation in elderly patients or different diseases other than urolithiasis. Their results showed that long-term vitamin D supplementation (a minimum supplementation period of 24 weeks) increased the risk of hypercalciuria, and the risk was not modified by Ca co-supplementation (24).

Another finding of our study was that although urinary Ca levels increased significantly, the risk of stone formation, which was assessed by CaOx SS and CaPSS, did not increase significantly. This is in line with the finding of Malihi et al. that although vitamin D supplementation increased the risk of hypercalciuria, it did not increase the risk of kidney stone formation (24). It should be kept in mind that a variety of urinary constituents may affect urine relative supersaturation (25). Thus, even if Ca rises because of vitamin D supplement, urine supersaturation could be normalized by increasing other inhibitor metabolites, such as Mg and Cit. Additional clinical trials on patients with lithiasis are needed to assess this finding.

Urine Ca is associated with different variables including dietary intake. Different dietary factors including a high intake of animal protein (26), Na (27, 28) and sucrose (17) could increase urine Ca. Our results showed that the level of urine Na (as a surrogate for Na intake) (16) and urea (as a surrogate for total protein intake) (26) increased simultaneously after vitamin D supplementation. Although there was no correlation between 24-h Na diff, 24-h Urea diff and 24-h Ca diff and, the simultaneous increase of Na and urea could be the cause for urine Ca increase. Moreover, other dietary factors, which could affect urine Ca, were not assessed in our study. Multivariate analyses of large clinical trials considering other confounding factors such as dietary information of patients could elucidate this effect.

The significant decrease in serum PTH was another noteworthy finding in our study. PTH is a hormone that regulates serum Ca levels through several different mechanisms, including increasing Ca reabsorption in renal tubules and mobilizing Ca from bones, which result in decreased bone density (8). The level of serum PTH rises in patients with vitamin D deficiency and should be decreased with vitamin D supplementation (8). Some references consider PTH reduction to be one of the targets for treating vitamin D deficiency (29). The significant decline of PTH in the current study could show the adequacy of vitamin D supplementation in our study. The changes in PTH were measured in studies by Leaf et al. (21) and Hesswani et al. (23). Interestingly, none of these two studies reported a significant change in serum PTH. As mentioned by the authors, the lack of PTH suppression could be due to inadequate changes in serum vitamin D levels after supplementation (23). The decrease in serum PTH may increase urinary Ca because of decreasing Ca reabsorption in renal tubules. However, our results did not show any correlation between urine Ca and serum PTH changes.

The response of serum PTH to vitamin D supplementation could be affected by different other factors such as Ca intake (30). Increasing Ca intake through diet or combined supplementation could increase PTH response, because PTH may not be suppressed without sufficient Ca intakes. All patients with recurrent calcium kidney stones should be advised to take 1000 to 1200mg of Ca per day through diet according to EAU guidelines (17), thus the significant change of PTH may be due in part to patient's sufficient Ca intake. However, we could not come to this conclusion due to the lack of calcium intake information. Undoubtedly, this should be assessed in other studies with dietary intake assessment.

Limitation of the current study, in addition to being retrospective, is the lack of control group, the possibility of over-collection, and the lack of data regarding dietary intakes of patients. However, the study had strict exclusion criteria including changes in dosage or new addition of thiazide diuretics, to rule out other factors, which may affect urinary Ca. Moreover, the study population was largely male which may reduce the generalizability of the results.

## CONCLUSIONS

In conclusion, the results of the current study suggest that although urine Ca increased in vitamin D supplemented patients, this increase was not associated with the increase in serum vitamin D, but may be secondary to other factors such as dietary Na intake. Further randomized clinical trials considering other factors associated with urine Ca, such as dietary intake of patients, are warranted.

## ABBREVIATIONS

Ca: = Calcium
25 (OH) D: = 25 hydroxyvitamin D
EAU: = European Association of Urology
PTH: = parathormone
Cr: = creatinine
Na: = sodium
Mg: = magnesium
Cit: = citrate
Ox: = oxalate
CaOxSS: = Supersaturations of CaOx
CaPSS: = Supersaturations of Ca phosphate
UASS: = Supersaturations of uric acid
24-h Ca: = 24-hour urine Ca
24-h Ca diff: = Changes in 24-h Ca
25(OH)D diff: = Changes in serum 25 (OH) D
PTH diff: = Changes in serum PTH
24-h Na: = 24-hour urine Na
24-h Urea: = 24-hour urine Urea
24-h Na diff: = Changes in 24-h Na
24-h Urea diff: = Changes in 24-h Urea
